# Editorial: Advances in Oscillating Reactions

**DOI:** 10.3389/fchem.2021.690699

**Published:** 2021-04-28

**Authors:** Željko D. Cupić, Annette F. Taylor, Dezso Horváth, Marek Orlik, Irving R. Epstein

**Affiliations:** ^1^Institute of Chemistry, Technology and Metallurgy, University of Belgrade, Belgrade, Serbia; ^2^Department of Chemical and Biological Engineering, The University of Sheffield, Sheffield, United Kingdom; ^3^Department of Applied and Environmental Chemistry, University of Szeged, Szeged, Hungary; ^4^Laboratory of Electroanalytical Chemistry, Faculty of Chemistry, University of Warsaw, Warsaw, Poland; ^5^Department of Chemistry, Brandeis University, Waltham, MA, United States

**Keywords:** non-linear dynamics, oscillating reactions, chemical computing, chaos, chemical oscillation

Numerous processes in physical chemistry and related sciences take place in a cyclic fashion, with time-periodic variations in concentrations widely known as oscillatory or oscillating reactions. Such processes have been explored as non-linear phenomena, but their potential in various applications became recognized only recently. Self-oscillating gels were developed to be used as components of micro devices (actuators) or to achieve controlled periodic drug delivery. Attempts were made to use oscillating reactions in detecting traces of CO in the air by special sensor technology. Also, a wide spectrum of analytical applications of oscillatory reactions was developed, with unexpected robustness and high sensitivity toward evaluation of pharmaceutically active compounds. Advantages of fuel cells in which electrocatalytic oxidations occur in an oscillating regime have also been reported.

The growth in applications of oscillatory reactions to a wide range of different areas has been enabled by new experimental techniques for both detection of exotic dynamic states and control of complex systems far from equilibrium. Also, new insights have been achieved in understanding the physical and chemical mechanisms underlying the extraordinary properties of such processes.

In the work by von Boehn and Imbihl, photoemission electron microscopy and low-energy electron microscopy were used to study the surface dynamics of ultrathin vanadium oxide layers epitaxially grown on Rh(111) and Rh(110) single crystal surfaces during catalytic methanol oxidation. Movement and coalescence of neighboring VO_x_ islands was observed, as well as phase separation inside the vanadium oxide islands, resulting in a substructure consisting of a reduced core and an outer oxidized ring, leading to periodic phase transitions and simultaneous size oscillations.

Shklyaev et al. determine how externally imposed flows affect chemical oscillations that are generated by two enzyme-coated patches within a fluid-filled millimeter-sized channel. An analytical model and simulations show that the distance between the enzyme-coated patches dictates the existence of chemical oscillations within the channel.

Li et al. generated both chemical oscillation and mechanical oscillation on lyotropic liquid crystalline gels, in which a Ru catalyst was embedded. Small fibers of gel were fabricated to achieve a macroscopically oscillatory bending–unbending transition driven by the Belousov–Zhabotinsky reaction.

Kurin-Csörgei et al. present results on new perborate oscillators. They successfully replaced H_2_O_2_ with NaBO_3_ in two oscillating reaction systems resulting in new ones: the ${\text{BO}}_{3}^{-}$ –S_2_${\text{O}}_{3}^{2-}$-Cu(II) and the ${\text{BO}}_{3}^{-}$ –SCN^−^-Cu(II) systems. Experimental measurements are supported by numerical simulations.

In the paper by Bubanja et al., intermittent forms of chaos are generated in the Bray-Liebhafsky oscillatory reaction, as a function of the specific flow rate in the CSTR. Chaotic complexity was observed in the vicinity of the critical control parameter value, between two extreme values for which periodic oscillations were observed. Although extremely hard to control, this type of dynamics has once more been confirmed as a robust phenomenon with a deterministic origin.

In the paper by Maria, glycolytic oscillations in *Escherichia coli* cells are subjected to *in silico* analysis to obtain a validated model useful for potential application. The model can be used to evaluate cellular metabolic fluxes, opening the possibility to re-design the cell metabolism *in silico* in order to obtain GMOs with industrial or medical applications.

The work of Fulczyk et al. provides an overview of studies on the hampering effect of heavy water (D_2_O) on the spontaneous oscillatory peptidization of selected proteinogenic α-amino acids. The experiments were carried out with the use of high-performance liquid chromatography, mass spectrometry, and scanning electron microscopy. These techniques were chosen to demonstrate spontaneous oscillatory peptidization of α-amino acids in the absence of D_2_O and the hampering effect of D_2_O on peptidization.

An extraordinarily important field for potential applications of oscillating reactions is chemical computing. Therefore, several authors have contributed to this issue with their results in this area. Muzika et al. examine dynamical switching among discrete Turing patterns in arrays of mass-coupled reaction cells. Carefully chosen transitions between discrete Turing patterns and uniform oscillations using precisely targeted perturbations were used to design chemical computing devices. Furthermore, computer simulations were used in the paper by Gorecki and Bose to explore the efficiency of chemical computing performed by a small network of three coupled chemical oscillators. The network was optimized to recognize the white and red regions of the Japanese flag. Finally, Perez-Mercader and Dueñas-Diez demonstrate that all physically realizable computing automata can be represented/assembled/built in the laboratory using oscillatory chemical reactions, and illustrate their implementation with the example of a programmable LBA Turing machine using the Belousov-Zhabotinsky oscillatory chemistry. Their device is capable of recognizing words in a Context Sensitive Language and rejecting words outside the language.

We hope that this overview of the latest work on non-linear dynamics in chemistry will inspire especially young researchers to further research in this fascinating field.

## Author Contributions

All authors listed have made a substantial, direct and intellectual contribution to the work, and approved it for publication.

## Conflict of Interest

The authors declare that the research was conducted in the absence of any commercial or financial relationships that could be construed as a potential conflict of interest.

